# Morphology, complete mitochondrial genome and molecular phylogeny of *Heterakis pucrasia* sp. n. (Nematoda: Ascaridida) from the koklass pheasant *Pucrasia macrolopha* (Lesson) (Galliformes: Phasianidae) in Pakistan

**DOI:** 10.3389/fvets.2025.1519092

**Published:** 2025-03-03

**Authors:** Muhammad Amjad Yousaf, Yi-Nuo Sun, Hui-Xia Chen, Asmat Ullah Khan, Liang Li

**Affiliations:** ^1^Hebei Collaborative Innovation Center for Eco-Environment, Hebei Key Laboratory of Animal Physiology, Biochemistry and Molecular Biology, College of Life Sciences, Hebei Normal University, Shijiazhuang, Hebei, China; ^2^Hebei Research Center of the Basic Discipline Cell Biology, Ministry of Education Key Laboratory of Molecular and Cellular Biology, Shijiazhuang, Hebei, China; ^3^Department of Zoology, Shaheed Benazir Bhutto University, Sheringal, Upper Dir, Khyber Pakhtunkhwa, Pakistan

**Keywords:** parasite, bird, Heterakoidea, integrative taxonomy, mitochondrial genome, phylogeny, new species

## Abstract

Species of *Heterakis* (Ascaridida: Heterakoidea) are commonest nematode parasites occurring in the alimentary canal of wild and domestic birds, which are of major socio-economic importance, due to some *Heterakis* species causing Heterakidosis in wild birds and poultry. In the present study, a new species of *Heterakis*, *H. pucrasia* sp. n., was described using integrated methods based on specimens collected from the koklass pheasant *Pucrasia macrolopha* (Lesson) (Galliformes: Phasianidae) in Pakistan. The complete mitochondrial genome of *H. pucrasia* sp. n. was sequenced and annotated for the first time to enrich the mitogenomic data, and reveal the pattern of mitogenomic evolution of the family Heterakidae. Moreover, phylogenetic analyses of the orders Ascaridida, Spirurida, Oxyurida and Rhigonematida based on the amino acid sequences of 12 protein coding genes (PCGs) of mitochondrial genomes, revealed that the order Ascaridida is not monophyletic, and the superfamily Heterakoidea has a closer affinity with Rhigonematida + Oxyurida + Spirurida, than the superfamily Ascaridoidea in Ascaridida. The present findings enriched the global species composition of heterakid nematodes and their mitogenomic data, and also provided novel insight on the phylogenetic relationships between Heterakoidea and its related groups.

## 1 Introduction

The koklass pheasant *Pucrasia macrolopha* (Lesson) (Galliformes: Phasianidae) is the only species of the monotypic genus *Pucrasia*, which is mainly distributed in China, Pakistan, India, Afghanistan and Nepal (1–3). The koklass pheasant is mostly herbivorous, mainly feeding on pine nuts, pine shoots, bamboo shoots, fruits and seeds, but it is highly insectivorous during the breeding season.^1^ However, our present knowledge of nematode parasites of the koklass pheasant remains very limited. To date, only *Heterakis gallinarum* (Schrank, 1788) has been reported from this bird (4).

The genus *Heterakis* (Ascaridida: Heterakoidea) is an important group of zooparasitic nematodes, including approximately 49 nominal species distributed globally, which mainly parasitize wild and domestic birds, but also occasionally occur in the alimentary canal of mammals (5–7). Some species of *Heterakis* (i.e., *H. gallinarum*, *H. dispar* and *H. isolonche*) can cause Heterakidosis in wild birds and poultry (8, 9). However, current knowledge of the global species composition of *Heterakis* nematodes remains far from complete. Furthermore, the molecular identification, population genetics and phylogeny of *Heterakis* spp. remain in their infancy (9–12), due to the limited genetic data available, especially the mitochondrial genomes. To date, only *H. gallinarum*, *H. beramporia* and *H. dispar* have been reported for the complete mitochondrial genomes in the genus *Heterakis* (13, 14).

In the present study, some *Heterakis* nematodes were collected from the koklass pheasant *P. macrolopha* in Pakistan. Multiple methods were used to study the specimens, including light and scanning electron microscopy, Assembled Species by Automatic Partitioning (ASAP) and Bayesian inference (BI) analyses, in order to exactly identify these specimens to species level. Moreover, the complete mitochondrial genome of the present material was sequenced and annotated to enrich the mitogenomic data and investigate the pattern of mitogenomic evolution of the family Heterakidae. Phylogenetic analyses were also performed in order to assess the systematic position of the superfamily Heterakoidea in the order Ascaridida, and the evolutionary relationships of the four orders Ascaridida, Spirurida, Oxyurida and Rhigonematida based on the amino acid sequences of 12 protein coding genes (PCGs) of mitogenomes using maximum likelihood (ML) and Bayesian inference (BI), respectively.

## 2 Methods

### 2.1 Parasite collection

During a helminthological survey of Pakistani birds in 2023, a single dead koklass pheasant *P. macrolopha* was dissected for parasites in the Sahoor Top Sheringal Upper Dir (35.2691° N, 72.0627° E), and a total of 23 nematode specimens were collected from the ceca of this bird. The specimens were fixed and stored in 80% ethanol until study.

### 2.2 Morphological observation

For light microscopy, nematode specimens were cleared in glycerin, then examined and photographed using a Nikon® optical microscope (Nikon ECLIPSE Ni-U, Nikon Corporation, Tokyo, Japan). For scanning electron microscopy (SEM), the anterior and posterior ends of 1 male and 1 female specimens, and the mid-body (vulval region) of 2 female specimens were transferred to 4% formaldehyde solution, post-fixed in 1% OsO₃, dehydrated via an ethanol series and acetone, and critical point dried. The specimens were coated with gold and examined using a Hitachi S-4800 scanning electron microscope (Hitachi Ltd., Tokyo, Japan) at an accelerating voltage of 20 kV. Measurements (minimum, maximum, followed by mean in parentheses) are given in micrometers (μm), unless otherwise stated. Type specimens were deposited at the College of Life Sciences, Hebei Normal University, Hebei Province, China.

### 2.3 Molecular procedures

The mid-body of three female specimens were randomly selected for molecular analysis. Genomic DNA from each individual was extracted using a Column Genomic DNA Isolation Kit (Shanghai Sangon, China) according to the manufacturer’s instructions, and stored at −20°C for further study. The primers and cycling conditions for amplifying the nuclear small ribosomal subunit (18S), large ribosomal subunit (28S) and internal transcribed spacer (ITS), and mitochondrial cytochrome c oxidase subunit 1 (*cox1*), cytochrome c oxidase subunit 2 (*cox2*) and small subunit ribosomal RNA gene (12S) by polymerase chain reaction (PCR) were provided in Supplementary Table S1. All PCR reactions were performed in 50 μL consisting of 10 mM Tris HCl at pH 8.4, 50 mM KCl, 3.0 mM MgCl₂, 250 μM of each dNTP, 50 pmol of each primer and 1.5 U of Taq polymerase (Takara Bio Inc., Kusatsu, Shiga, Japan) in a thermocycler (model 2,720; Applied Biosystems, Thermo Fisher Scientific, Waltham, MA, USA). PCR products were checked on GoldView-stained 1.5% agarose gel and purified using the Column PCR Product Purification Kit (Shanghai Sangon, China). Sequencing of each sample was carried out for both strands using a DyeDeoxyTerminator Cycle Sequencing Kit (v.2, Applied Biosystems, California, USA). The ITS1-5.8S-ITS2 region was assembled using GetOrganelle v1.7.7.0 (15) based on genomic data sequenced by Illumina NovaSeq 6,000 platform. The 18S, 28S, ITS, *cox1*, *cox2* and 12S sequences obtained herein were deposited in the National Center for Biotechnology Information (NCBI) database.^2^

### 2.4 Species delimitation

The Assembled Species by Automatic Partitioning (ASAP) (16) was used for species delimitation of *Heterakis* spp. based on different genetic markers (i.e., ITS, *cox1*, *cox2,* and 12S sequences). *Ascaridia columbae* was chosen as the out-group. The ASAP analyses were performed using an online ASAP web server^3^ under the Kimura (k8o) ts/tv 2.0 model. The group with the lowest score from ASAP was considered to be optimal.

### 2.5 Mitochondrial genome sequencing, assembly, and annotation

The mid-body of 1 female specimen was used for mitogenome sequencing. A total of 50 GB of clean genomic data was generated using the Pair-End 150 sequencing method on the Illumina NovaSeq 6,000 platform by Novogene (Tianjin, China). The complete mitochondrial genome was assembled using GetOrganelle v1.7.7.0 (15). Protein coding genes (PCGs), rRNAs and tRNAs were annotated using the MITOS2 web server^4^ and MitoZ v3.6 (17). The open reading frame (ORF) of each PCG was confirmed manually using the web version of the ORF finder.^5^ The “lost” tRNA genes ignored by both MitoS2 and MitoZ (17), were identified using BLAST-based on a database of existing tRNA sequences of nematodes. The secondary structures of tRNAs were predicted using ViennaRNA module (18), by MitoS2 and RNAstructure v6.4 (19), followed by manual correction. MitoZ v3.6 was used to visualize and depict gene element features (17). Base composition, amino acid usage, and relative synonymous codon usage (RSCU) were calculated using Python script, which refers to the Codon Adaptation Index (CAI) (20). The total length of the bases included ambiguous bases. Base skew analysis was used to describe the base composition. The complete mitochondrial genome obtained herein was deposited in the GenBank database (see footnote 2).

### 2.6 Phylogenetic analyses

Phylogenetic analyses of *Heterakis* spp. were performed based ITS, *cox1*, *cox2* and 12S sequence data, using Bayesian inference (BI) with MrBayes v3.2.7a (21). *Ascaridia columbae* was chosen as the out-group. Phylogenetic analyses of Ascaridida, Spirurida, Oxyurida and Rhigonematida were conducted based on concatenated amino acid (AA) sequences of the 12 PCGs using maximum likelihood (ML) with IQ-TREE v2.2.2.7 (22), and Bayesian inference (BI) with MrBayes v3.2.7a (21). *Caenorhabditis elegans* (Rhabditida: Rhabditidae) was chosen as the outgroup. The ingroup included 56 species representing 11 superfamilies of these four orders. Detailed information on the representatives of Ascaridida, Spirurida, Oxyurida and Rhigonematida included in the present phylogenetic analyses was provided in Supplementary Table S2. Genes were aligned separately with MAFFT v7.526 using the iterative refinement method of E-INS-I (23). Ambiguous sites and poorly aligned positions were eliminated using BMGE v1.12 (m = BLOSUM90, h = 0.5) (24). The aligned and eliminated sequences were concatenated into a matrix using PhyloSuite v1.2.3 (25). The mtMet+F+R4 model for ML inference, and the JTT+I+G model for BI were identified as the optimal nucleotide substitution models, respectively. Reliabilities for ML inference was tested using 1,000 bootstrap replications and Bayesian information criterion for BI analysis was run for 5 × 10^6^ Markov Chain Monte Carlo (MCMC) generations. In the ML tree, bootstrap support (BS) values ≥90 were considered to constitute strong nodal support, whereas BS values ≥70 and < 90 were considered to constitute moderate nodal support. In the BI tree, Bayesian posterior probability (BPP) values ≥0.90 were considered to constitute strong nodal support, whereas BPP values≥0.85 and < 0.90 were considered to constitute moderate nodal support.

## 3 Results

### 3.1 Description of *Heterakis pucrasia* sp. n. (Figures 1–4)

**Figure 1:**
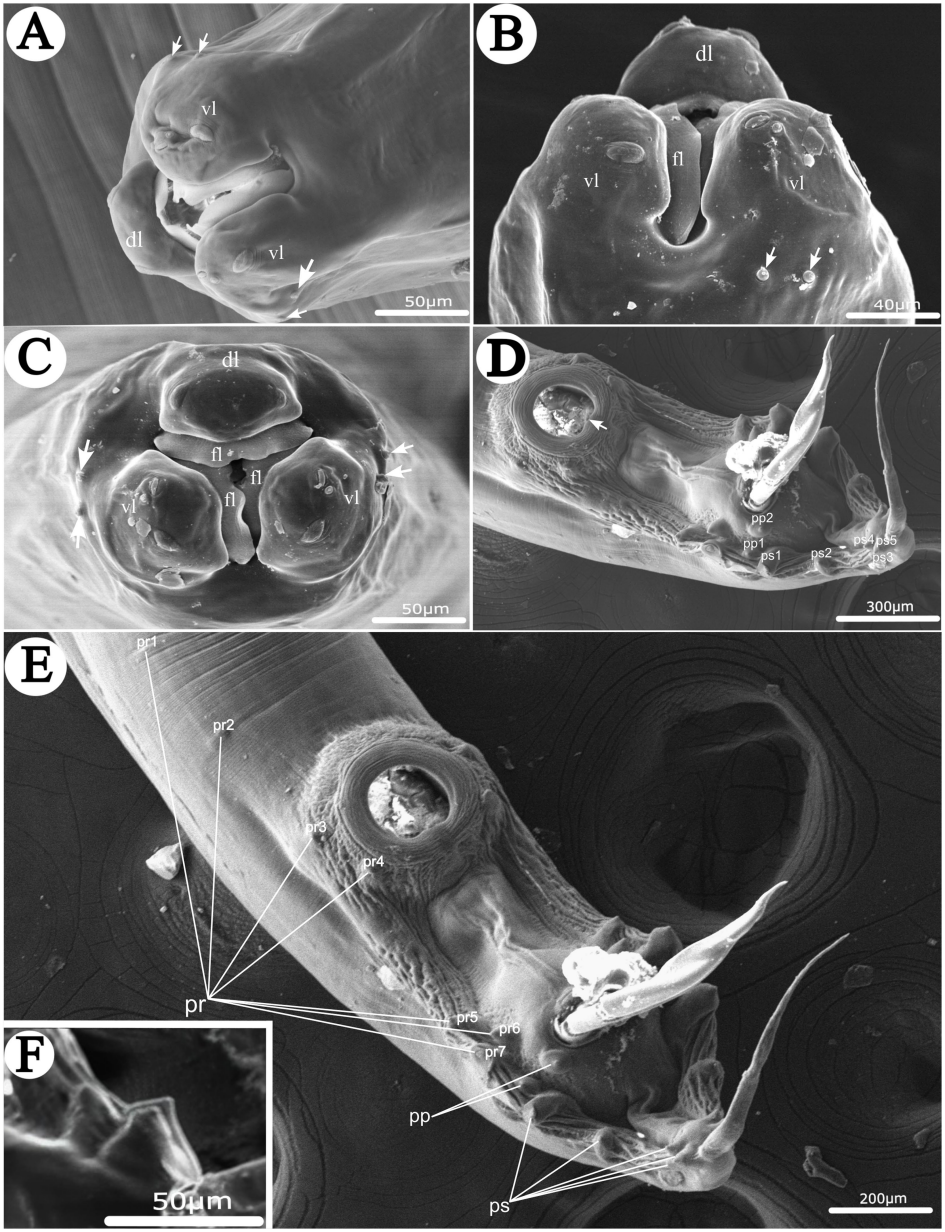
Scanning electron micrographs of *Heterakis pucrasia* sp. n. from *Pucrasia macrolopha* in Pakistan. **(A)** Cephalic extremity of male (cephalic papillae arrowed), subapical view. **(B)** Cephalic extremity of female (cephalic papillae arrowed), ventral view. **(C)** Cephalic extremity of female (cephalic papillae arrowed), apical view. **(D)** Posterior end of male (single medio-ventral sessile papilla on posterior margin of precloacal sucker arrowed), ventral view. **(E)** Posterior end of male, ventral view. **(F)** Magnified image of last 3 pairs of postcloacal papillae. dl, dorsal lip; vl, ventrolateral lip; fl, inner flanges on each lip; pr, precloacal papillae; pp., paracloacal papillae; ps, postcloacal papillae.

**Figure 2:**
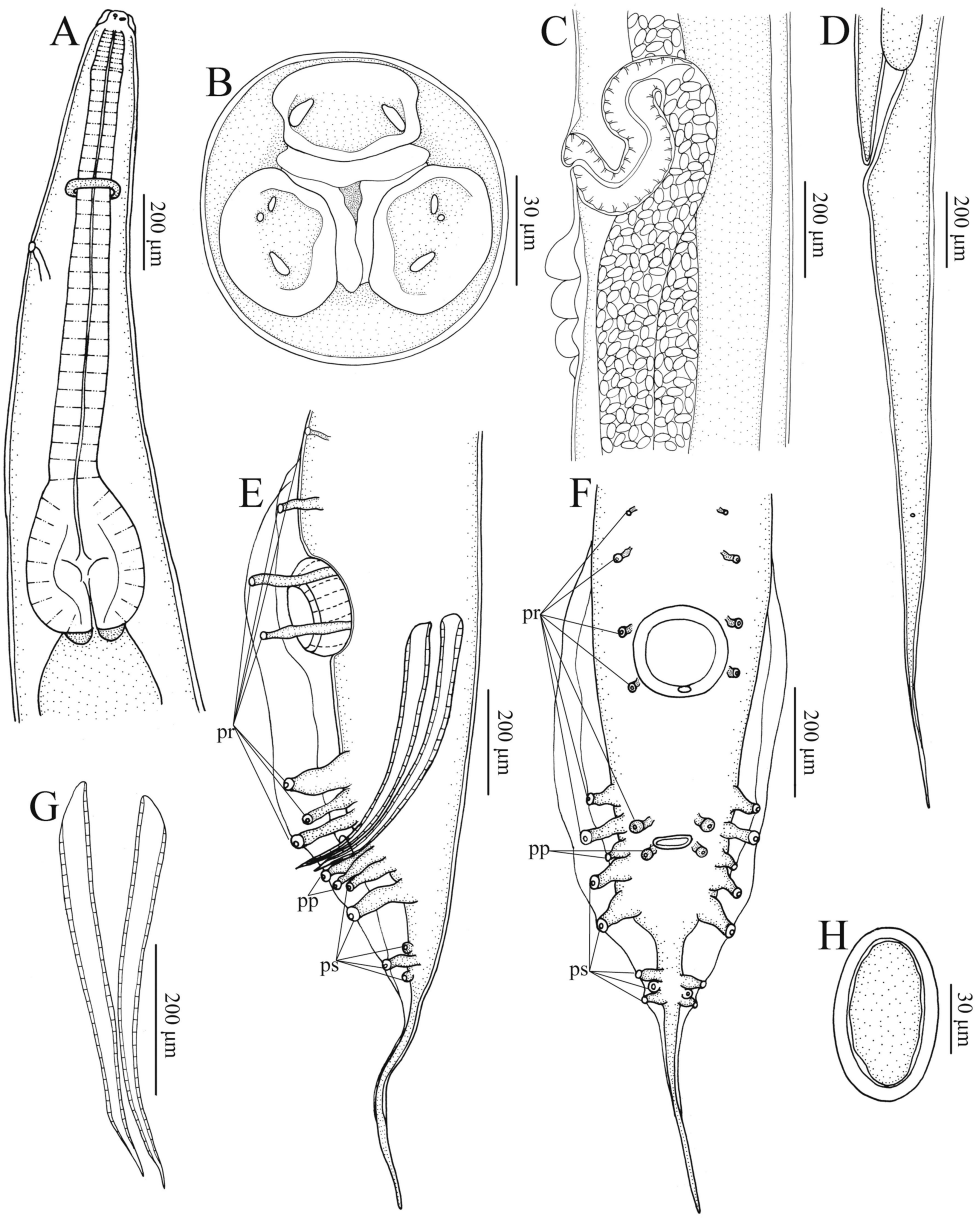
*Heterakis pucrasia* sp. n. from *Pucrasia macrolopha* in Pakistan. **(A)** Anterior part of female, sublateral view. **(B)** Cephalic extremity of female, apical view. **(C)** Region of vulva, lateral view. **(D)** Tail of female, lateral view. **(E)** Posterior end of male, lateral view. **(F)** Posterior end of male, ventral view. **(G)** Spicules. **(H)** Egg. pr, precloacal papillae; pp., paracloacal papillae; ps, postcloacal papillae.

**Figure 3:**
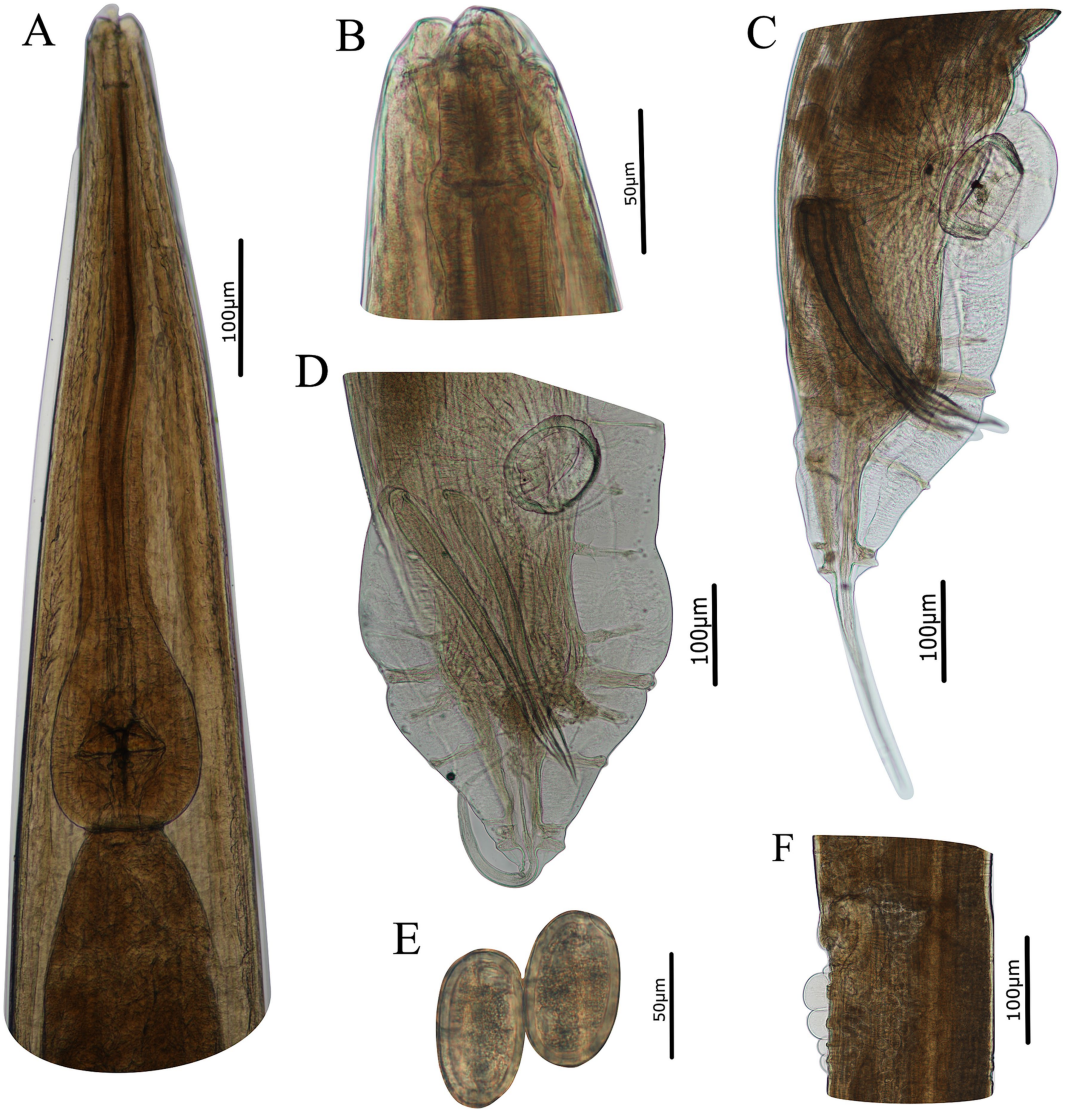
Photomicrographs of *Heterakis pucrasia* sp. n. from *Pucrasia macrolopha* in Pakistan. **(A)** Anterior part of female, ventral view. **(B)** Cephalic extremity of female, sublateral view. **(C)** Posterior end of male, lateral view. **(D)** Posterior end of male, ventral view. **(E)** Eggs. **(F)** Region of vulva, lateral view.

**Figure 4:**
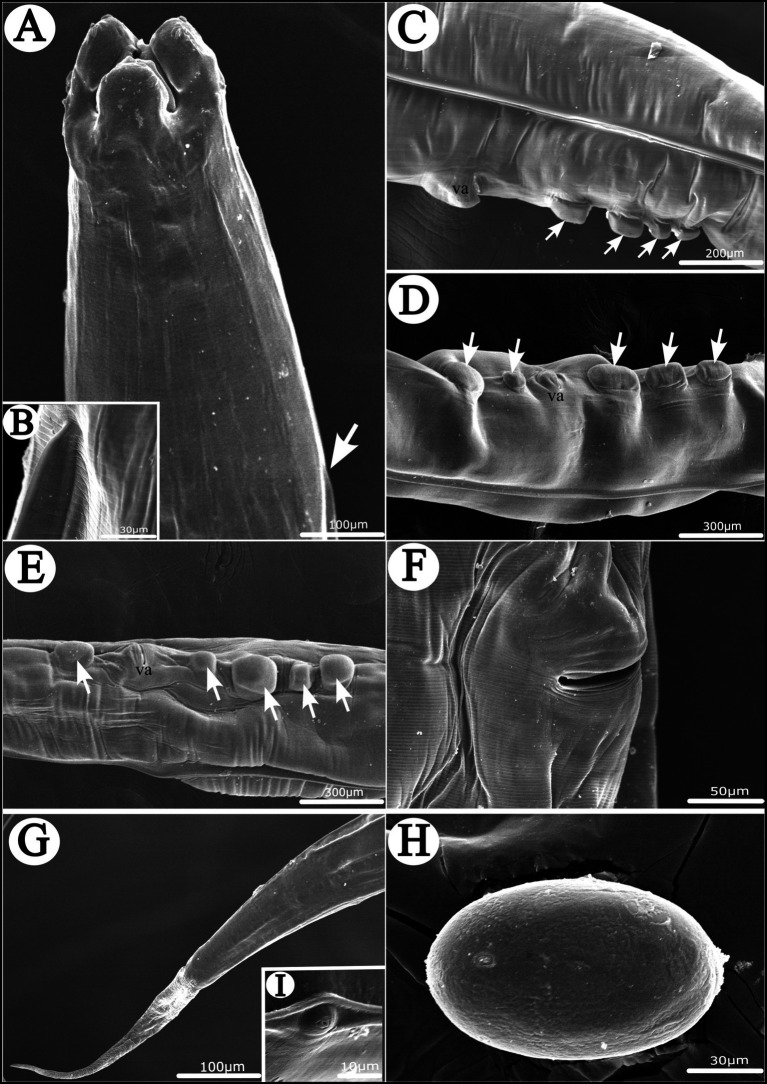
Scanning electron micrographs of *Heterakis pucrasia* sp. n. from *Pucrasia macrolopha* in Pakistan, female. **(A)** Anterior part of body (starting position of lateral ala arrowed), dorsal view. **(B)** Magnified image of starting position of lateral ala. **(C–E)** Region of vulva showing different number and arrangement of cuticular protrusions at region of vulva (cuticular protrusions arrowed), lateral or subventral view. **(F)** Magnified image of vulva. **(G)** Tail tip. **(H)** Magnified image of egg. **(I)** Magnified image of phasmid. va, vulva.

*General*: Medium-sized, whitish nematodes with finely transversely striated cuticle. Maximum width at about region of mid-body. Cephalic extremity with three roughly trapeziform lips, approximately equal in size; each lip possesses remarkable inner flanges (Figures 1A–C, 2A,B, 3A,B, 4A). Dorsal lip with one pair of large double labial papillae; ventrolateral lips each with single double labial papilla, small labial papilla, and amphid (Figures 1A–C, 2B, 3A). One pair of cephalic papillae present at base of each ventro-lateral lip (Figures 1A–C). Interlabia absent. Esophagus muscular, divided into anterior short pharynx, cylindrical corpus, inconspicuous isthmus and terminating posterior bulb with valves (Figures 2A, 3A,B). Nerve ring at about 1/5 of esophageal length. Excretory pore just posterior to nerve ring (Figure 2A). Lateral alae well-developed, starting from some distance posterior to base of ventrolateral lips extending to caudal region (Figures 3A, 4A,B). Tails of both sexes conical, with long filamentous tips (Figures 1D–F, 3C,D, 4G).

*Male* [based on 2 mature specimens] Body 9,100–9,390 (9,245 ± 145) long, maximum width 480–510 (495 ± 15). Dorsal and ventrolateral lips almost equal in size, 24–29 (27 ± 2) long, 53–58 (56 ± 2) wide. Esophagus 1,370–1,420 (1,395 ± 25) long in total length (including posterior bulb), representing 10.7–11.7 (11.2) % of body length; pharynx + corpus + isthmus 1,000–1,060 (1,030 ± 30) long, size of posterior bulb 360–370 (365 ± 5) × 290–300 (295 ± 5). Nerve-ring and excretory pores 390–390 (385 ± 5) and 550–680 (615 ± 65) from the cephalic extremity, respectively. Posterior end of body distinctly curves ventrally. Spicules short, alate, nearly equal in length, distal end pointed, 550–570 (560 ± 10) long, representing 5.80–6.26 (6.06) % of body length (Figures 1D,E, 2E,G, 3C,D). Gubernaculum absent. Precloacal sucker well developed, circular, 190–200 (195 ± 5) in diameter, 310–350 (330 ± 20) from its center to cloaca; single medio-ventral sessile papilla present at posterior margin of precloacal sucker (Figures 1D,E, 2E,F, 3C,D). Caudal papillae 14 pairs in total, including 7 pairs of precloacal papillae (1st pair small, sessile; 2nd pair being pedunculate, located at about starting position of caudal alae; 3rd and 4th pairs being pedunculate, located at about level of anterior and posterior margin of sucker, respectively; 5th–7th pairs large, close to each, slightly anterior to cloaca, 5th and 6th pairs located ventrolaterally, being pedunculate, 7th pair located ventrally, being sessile), 2 pairs of paracloacal papillae (one pair located ventrolaterally, being pedunculate; the other located ventrally, being sessile) and 5 pairs of postcloacal pedunculate papillae (anterior 2 pairs large; posterior 3 pairs small, grouped together) (Figures 1D–F, 2E,F, 3C,D). Tail 650–660 (655 ± 5) long (Figures 1D–F, 2E,F, 3C,D). Phasmids not observed.

*Female* [based on 10 gravid specimens] Body 10,317–14,220 (12,135 ± 1,527) long, maximum width 420–560 (500 ± 41). Dorsal and ventrolateral lips almost equal in size, 24–39 (32 ± 4) long, 48–58 (55 ± 3) wide. Esophagus 1,430–1,600 (1,532 ± 56) long in total length (including posterior bulb), representing 11.3–13.9 (12.6) % of body length; pharynx + corpus + isthmus 1,010–1,220 (1,152 ± 59) long, size of posterior bulb 280–310 (296 ± 8) × 280–310 (296 ± 8). Nerve ring 370–430 (399 ± 21) and excretory pore 580–680 (619 ± 27) from cephalic extremity. Vulva slit-like, with slightly protruded vulval lip (Figures 2C, 3F, 4C–F), 4,470–6,995 (5,860 ± 907) from cephalic extremity, representing 43.3–53.5 (48.2) % of body length. Four or five cuticular protrusions located at region of vulva in various arrangement types (Figures 2C, 3F, 4C–E). Vagina muscular, uteri didelphic, opistodelphic. Eggs oval, thick-shelled, 68–72 (71 ± 2) × 39–43 (43 ± 1) (n = 20) in size (Figures 2H, 3E, 4H). Tail 1,310–1,680 (1,517 ± 128) long (Figures 2D, 3G). Phasmids at about posterior 1/3 of the tail (Figures 2D, 4I).

*Type host*: Koklass pheasant *Pucrasia macrolopha* (Lesson) (Galliformes: Phasianidae).

*Type locality*: Sahoor Top Sheringal Upper Dir (35.2691° N, 72.0627° E), Pakistan.

*Site in host*: Ceca.

*Prevalence and intensity*: Single koklaas pheasant examined with an intensity of 23 specimens.

*Type specimens*: Holotype: 1 male (HBNU–N–B20240426YL); allotype: 1 female (HBNU–N–B20240427YL); paratypes: 1 complete male and 12 complete females (HBNU–N–R20240428YL), 1 incomplete male (only anterior and posterior end, mid-body for molecular analysis) and 7 incomplete females (only anterior and posterior end, mid-body for molecular analysis) (HBNU–N–B20240428YL); deposited in the College of Life Sciences, Hebei Normal University, Hebei Province, China.

To comply with the regulations set out in article 8.5 of the amended 2012 version of the International Code of Zoological Nomenclature (ICZN), details of the new species have been submitted to ZooBank. The Life Science Identifier (LSID) of the article is urn:lsid:zoobank.org:pub:C5319C09-F4DB-4CCA-99C6-02DE2B88B41C. The LSID for the new name Heterakis pucrasia sp. n. is urn:lsid:zoobank.org:act:9CAFD43C-072A-4294-A06B-AC97025AC838.

*Etymology*: The specific name refers to the generic name of the host type.

### 3.2 Genetic characterization

#### 3.2.1 Partial 18S region

Three 18S sequences of *H. pucrasia* sp. n. obtained herein are 781 bp long, with no nucleotide divergence detected. In the genus *Heterakis*, the 18S sequences are available in GenBank for *H. gallinarum* (DQ503462, MK844591, MW073433, MW073432 and MW073553), *Heterakis* sp. (AF083003) and *H. spumosa* (MH571872). Pairwise comparison of the 18S sequences of *H. pucrasia* sp. n. with that of *H. gallinarum*, *Heterakis* sp. and *H. spumosa* available in GenBank showed 0.40% (*H. gallinarum*) to 5.72% (*Heterakis* sp.) of nucleotide divergence.

#### 3.2.2 Partial 28S region

Three 28S sequences of *H. pucrasia* sp. n. obtained herein are 1,071 bp long, with no nucleotide divergence detected. In the genus *Heterakis*, only *H. gallinarum* (LC777441) and *H. spumosa* (MH571869) with the partial 28S sequence are available in GenBank. Pairwise comparison of the 28S sequences of *H. pucrasia* sp. n. with that of *H. gallinarum* and *H. spumosa* available in GenBank showed 11.1% (*H. gallinarum*) to 11.3% (*H. spumosa*) of nucleotide divergence.

#### 3.2.3 Partial ITS (ITS-1+5.8S+ITS-2) region

Single ITS sequence of *H. pucrasia* sp. n. obtained herein is 954 bp long. In the genus *Heterakis*, the ITS sequences are available in GenBank for *H. gallinarum* (MW408729–MW412732, MW403874, MW661165, MW561275, MF066721–MF066726, MF403056, LC592776–LC592805, LC592808, KT310099–KT310157, OQ848045, JQ995320, OR619556, AJ876757), *H. indica* (LC592806–LC592809), *H. dispar* (OM530142–OM530145, OM530147, MF319969), *H. isolonche* (KM212953), *Heterakis* sp. (ON244646–ON244647), *H. spumosa* (LC389877, JX845278), *H. beramporia* (LC592731–LC592733, KU529974, OL470970–OL470973) and *H. dahomensis* (JX845277). Pairwise comparison of the ITS sequence of *H. pucrasia* sp. n. with that of *Heterakis* spp. available in GenBank showed 8.47–8.82% (*H. dispar*) to 12.2–18.2% (*H. beramporia*) of nucleotide divergence.

#### 3.2.4 Partial *cox1* region

Three *co*x1 sequences of *H. pucrasia* sp. n. obtained herein are 682 bp long, with no nucleotide divergence detected. In the genus *Heterakis*, the *cox1* sequences are available in GenBank for *H. gallinarum* (KP308308–KP308363, MF066712–MF066720, LC592865, NC_029839, KU529973), *H. indica* (LC592869–LC592874), *H. dispar* (NC_042411, MK024389), *H. isolonche* (MN732844–MN732847, FJ009625–FJ009627), *Heterakis* sp. (MN737038–MN737040, MN732849–MN732852), *H. spumosa* (LC626017) and *H. beramporia* (LC592900–LC592902, NC_029838, KU529972). Pairwise comparison of the *cox1* sequence of *H. pucrasia* sp. n. with that of *Heterakis* spp. available in GenBank showed 9.20% (*H. gallinarum*) to 18.1% (*H. isolonche*) nucleotide divergence.

#### 3.2.5 Partial *cox2* region

Three *cox2* sequences of *H. pucrasia* sp. n. obtained herein are 519 bp long, with no nucleotide divergence detected. In the genus *Heterakis*, the *cox2* sequences are available in GenBank for *H. gallinarum* (MF114536–MF114591, NC_029839), *H. beramporia* (NC_029838) and *H. dispar* (NC_042411). Pairwise comparison of the *cox2* sequences of *H. pucrasia* sp. n. with that of *Heterakis* spp. available in GenBank showed 10.6–11.0% (*H. gallinarum*) to 11.2% (*H. dispar*) nucleotide divergence.

#### 3.2.6 Partial 12S region

Three 12S sequences of *H. pucrasia* sp. n. obtained herein are 486 long, with no nucleotide divergence. In the genus *Heterakis*, the 12S sequences are available in GenBank for *H. gallinarum* (KR080329, KR153881–KR153937, NC_029839.1), *H. dispar* (NC_042411), *H. beramporia* (NC_029838) and *H. spumosa* (MT135066, MT135065). Pairwise comparison of the 12S sequences of *H. pucrasia* sp. n. with that of *Heterakis* spp. available in GenBank showed 8.27% (*H. gallinarum*) to 13.5–13.6% (*H. spumosa*) nucleotide divergence.

### 3.3 Species delimitation

**Figure 5:**
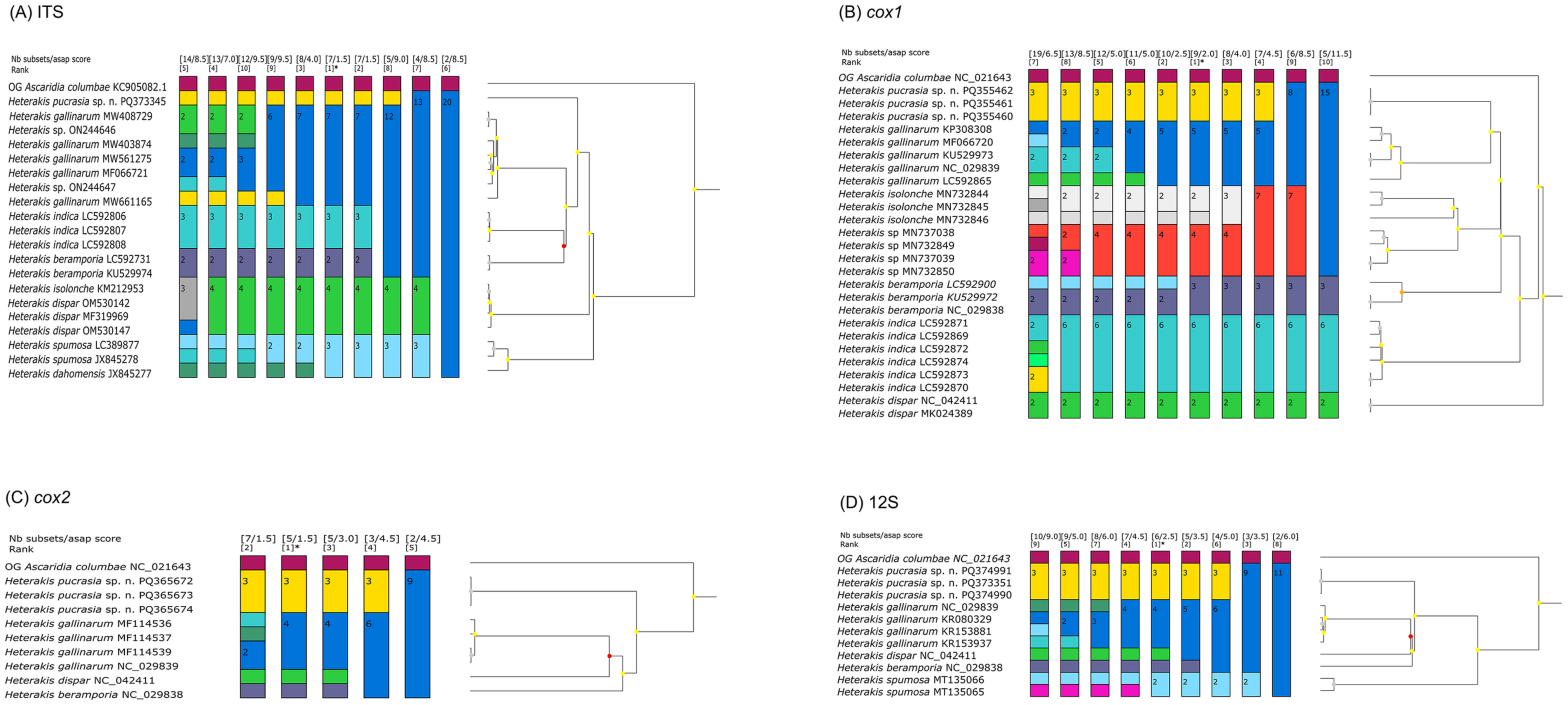
Assemble species by automatic partitioning (ASAP) analyses of *Heterakis* spp. based on different genetic markers. Asterisk representing the optimal result recommended by ASAP. *Ascaridia columbae* was chosen as the out-group (OG). **(A)** ASAP results based on ITS data. **(B)** ASAP results based on *cox1* data. **(C)** ASAP results based on *cox2* data. **(D)** ASAP results based on 12S data.

**Figure 6:**
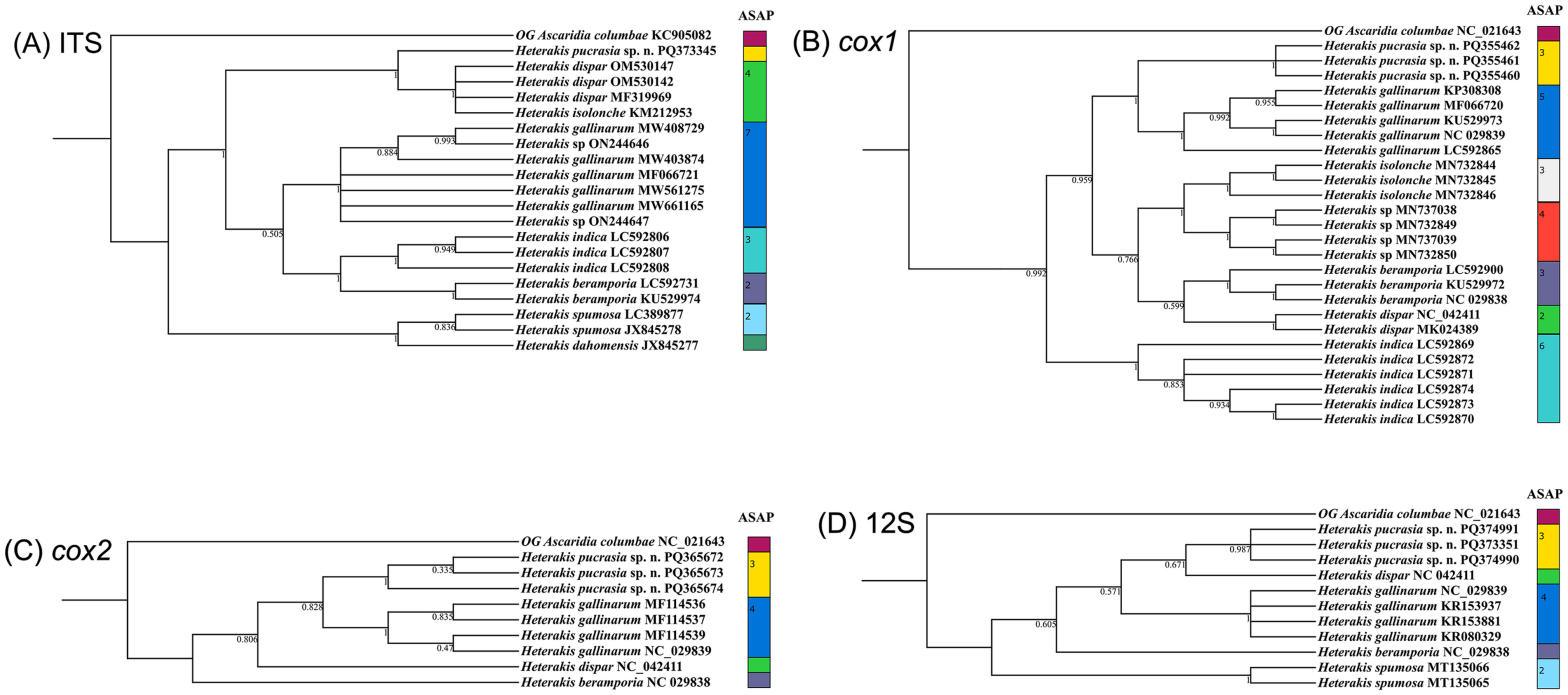
Comparative results of Bayesian inference (BI) and ASAP of *Heterakis* spp. based on different genetic markers. *Ascaridia columbae* was chosen as the out-group (OG). **(A)** BI results based on ITS data. **(B)** BI results based on *cox1* data. **(C)** BI results based on *cox2* data. **(D)** BI results based on 12S data.

The results of ASAP and BI analyses based on the ITS, *cox1*, *cox2* and 12S sequences, all supported that *H. pucrasia* sp. n. represents a separated species from its congeners (Figures 5, 6). However, the optimal results of ASAP and BI analyses based on the ITS sequences did not support the species partition of *H. dispar* and *H. isolonche*, and *H. spumosa* and *H. dahomensis* (Figure 5). The results of BI analyses based on the ITS and 12S sequences showed that *H. pucrasia* sp. n. is a sister to *H. dispar*, but the BI results based on the *cox1* and *cox2* sequences displayed *H. pucrasia* sp. n. and *H. gallinarum* have a closer relationship than *H. dispar* (Figure 6).

### 3.4 Characterization of complete mitochondrial genome

**Figure 7:**
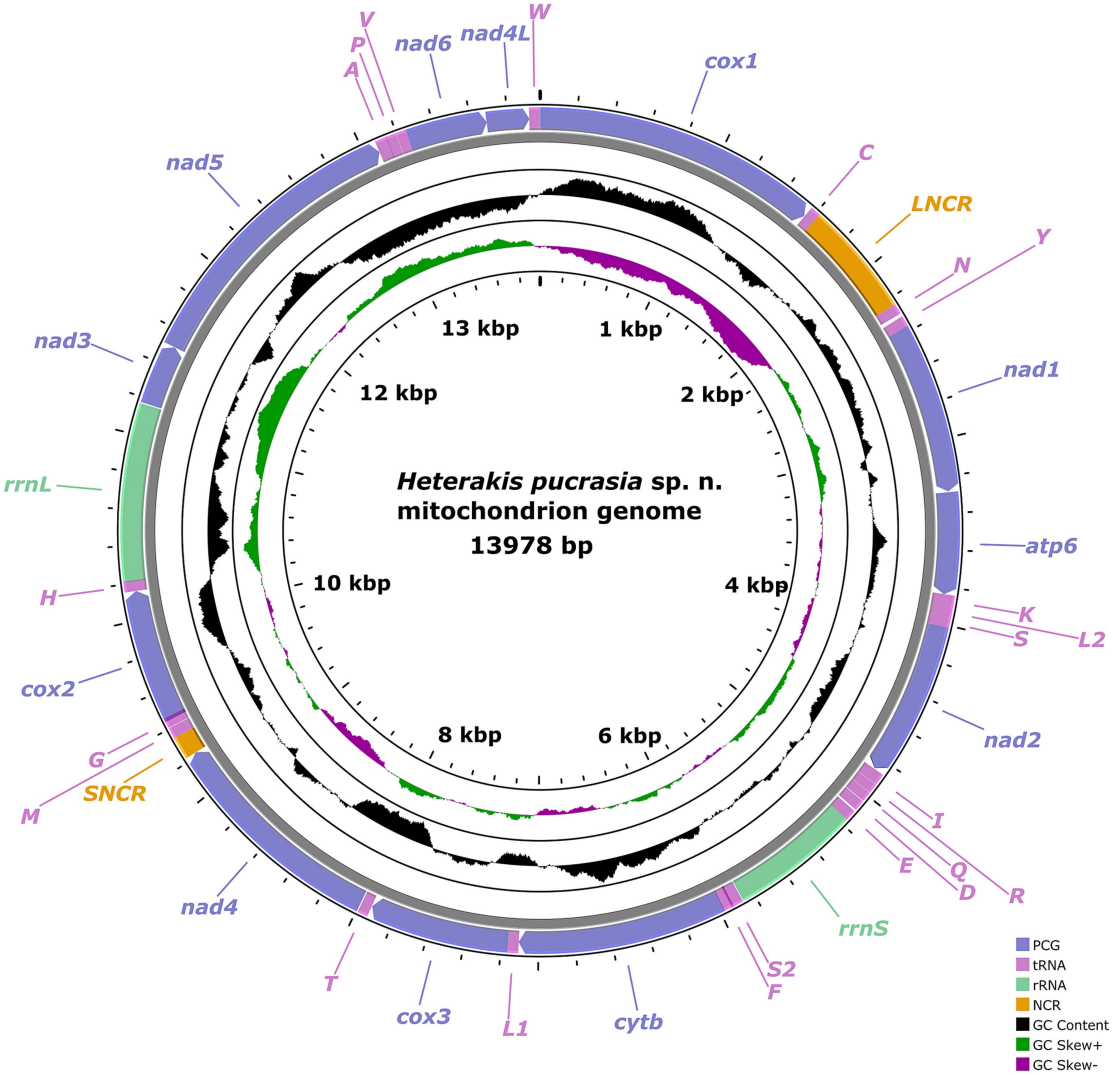
Gene map of the mitochondrial genome of *Heterakis pucrasia* sp. n. All 22 tRNA genes are nominated by the one-letter coding with numbers differentiating each of the two tRNAs, serine and leucine.

The mitogenome of *H. pucrasia* sp. n. is 13,978 bp in length, containing 36 genes, including 12 PCGs (missing *atp8*) (*cox1–3*, *cyt*b, *nad*1–6, *nad4*L and *atp*6), 22 tRNA genes and 2 rRNA genes (*rrnL* and *rrnS*) (Figure 7; Supplementary Table S1). All genes are transcribed from the same DNA strand. Two non-coding regions (LNCR and SNCR) are present in the mitogenome of *H. pucrasia* sp. n. (LNCR is 619 bp, between *tRNA-Cys* and *tRNA-Asn*; SNCR is 123 bp, between *nad4* and *tRNA-Met*) (Figure 7). The overall A+T content in the mitogenome of *H. pucrasia* sp. n. is 68.9%, displaying a strong nucleotide compositional bias toward A+T (Supplementary Table S2). The nucleotide content of the mitogenome of *H. pucrasia* sp. n. was provided in Supplementary Table S2.

**Figure 8:**
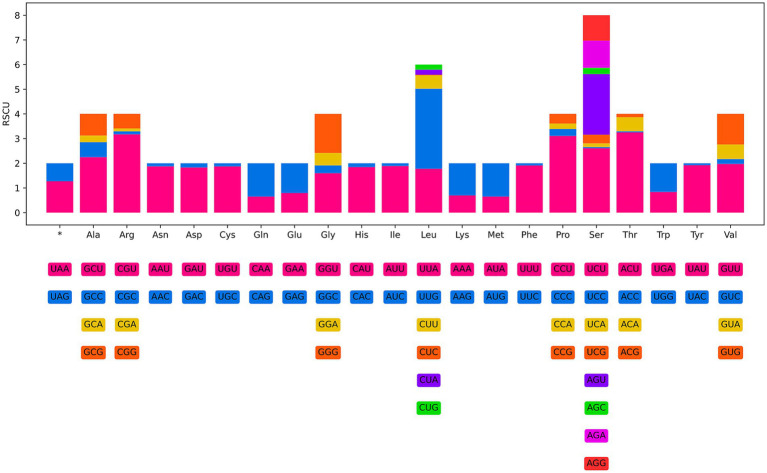
Relative synonymous codon usage (RSCU) of *Heterakis pucrasia* sp. n. The codon families (in alphabetical order) indicated below the horizontal axis. Values at the top of each bar representing amino acid usage in percentage.

The 12 PCGs of the mitogenome of *H. pucrasia* sp. n. has 10,277 bp (excluding termination codons), ranged in size from 226 bp (*nad4L*) to 1,563 bp (*cox1*), which encoded 3,427 amino acids. Among the 12 PCGs, TTG is the most common start codon used for five genes (*nad1*, *nad2*, *nad3, nad5* and *nad6*), followed by ATT for three genes (*cox2*, *cox3* and *nad4L*), and ATG for *cox1* and *cytb* and ATA for atp*6* and *nad*4. TAA is the most commonly used termination codon for seven genes (*cox3*, *nad1*, *nad2*, *nad4, nad5, nad6*, and *cytb*), followed by TAG for *cox1*, *nad3, cox2* and *atp6*, and the incomplete stop codon TA for *nad4L* (Table 1). The components and usage of codons in the mitogenome of *H. pucrasia* sp. n. were provided in Table 1 and Figure 8. The length of 22 tRNAs in the mitogenome of *H. pucrasia* sp. n. was provided in Table 1.

**Table 1:** Annotations and gene organization of mitogenome of *Heterakis pucrasia* sp. n. (PQ389430).

Gene	Type (bp)	Start (bp)	End (bp)	Length (bp)	Start codon	Termination codon	Anticodon	Strand	Gap or overlap
*cox1*	CDS	1	1,563	1,563	ATG	TAG		+	2
*tRNA-Cys*	tRNA	1,566	1,620	55			GCA	+	0
LNCR	NCR	1,621	2,239	618				+	0
*tRNA-Asn*	tRNA	2,240	2,296	57			GUU	+	16
*tRNA-Tyr*	tRNA	2,313	2,369	57			GUA	+	-2
*nad1*	CDS	2,368	3,243	876	TTG	TAA		+	0
*atp6*	CDS	3,244	3,840	597	ATA	TAG		+	0
*tRNA-Lys*	tRNA	3,841	3,904	64			UUU	+	−1
*tRNA-Leu2*	tRNA	3,904	3,958	55			UAA	+	0
*tRNA-Ser1*	tRNA	3,959	4,011	53			UCU	+	6
*nad2*	CDS	4,018	4,857	840	TTG	TAA		+	7
*tRNA-Ile*	tRNA	4,865	4,927	63			GAU	+	1
*tRNA-Arg*	tRNA	4,929	4,980	52			CGG	+	1
*tRNA-Gln*	tRNA	4,982	5,036	55			UUG	+	3
*tRNA-Asp*	tRNA	5,040	5,097	58			GUC	+	10
*tRNA-Glu*	tRNA	5,108	5,167	60			UUC	+	0
*rrnS*	rRNA	5,168	5,864	697				+	4
*tRNA-Ser2*	tRNA	5,869	5,927	59			UGA	+	-5
*tRNA-Phe*	tRNA	5,923	5,979	57			GAA	+	-3
*cytb*	CDS	5,977	7,107	1,131	ATG	TAA		+	-1
*tRNA-Leu1*	tRNA	7,107	7,164	58			UAG	+	0
*cox3*	CDS	7,165	7,932	768	ATT	TAA		+	2
*tRNA-Thr*	tRNA	7,935	7,990	56			UGU	+	6
*nad4*	CDS	7,997	9,220	1,224	ATA	TAA		+	0
SNCR	SNCR	9,221	9,343	123				+	0
*tRNA-Met*	tRNA	9,344	9,403	60			CAU	+	2
*tRNA-Gly*	tRNA	9,406	9,460	55			UCC	+	−21
*cox2*	CDS	9,440	10,156	717	ATT	TAG		+	1
*tRNA-His*	tRNA	10,158	10,215	58			GUG	+	−3
*rrnL*	rRNA	10,213	11,166	954				+	9
*nad3*	CDS	11,176	11,532	357	TTG	TAG		+	1
*nad5*	CDS	11,534	13,075	1,542	TTG	TAA		+	4
*tRNA-Ala*	tRNA	13,080	13,143	64			UGC	+	−2
*tRNA-Pro*	tRNA	13,142	13,197	56			UGG	+	1
*tRNA-Val*	tRNA	13,199	13,253	55			UAC	+	0
*nad6*	CDS	13,254	13,688	435	TTG	TAA		+	−1
*nad4L*	CDS	13,688	13,918	231	ATT	TA		+	1
*tRNA-Trp*	tRNA	13,920	13,977	58			UCA	+	2

Positive number in the “Gap or overlap” column indicates the length of intergenic sequence, and the negative number indicates the length (absolute number) that adjacent genes overlap. (negative sign). The forward strand is marked as “+” and the reverse strand as “-”.

The 36 gene arrangements in the mitogenome of *H. pucrasia* sp. n. are in the following order: *cox1*, *tRNA-Cys*, *tRNA-Asn*, *tRNA-Tyr*, *nad1*, *atp6*, *tRNA-Lys, tRNA-Leu2, tRNA-Ser1, nad2*, *tRNA-Ile*, *tRNA-Arg*, *tRNA-Gln*, *tRNA-A*sp.*, tRNA-Glu*, *rrnS, tRNA-Ser2*, *tRNA-Phe*, *cyt*b, *tRNA-Leu1*, *cox3*, *tRNA-Thr*, *nad4*, *tRNA-Met*, *tRNA-Gly*, *cox2*, *tRNA-His*, *rrnL*, *nad3*, *nad5*, *tRNA-Ala, tRNA-Pro*, *tRNA-Val, nad6*, *nad4L*, and *tRNA-Trp* (Figure 7).

### 3.5 Phylogenetic analyses

**Figure 9:**
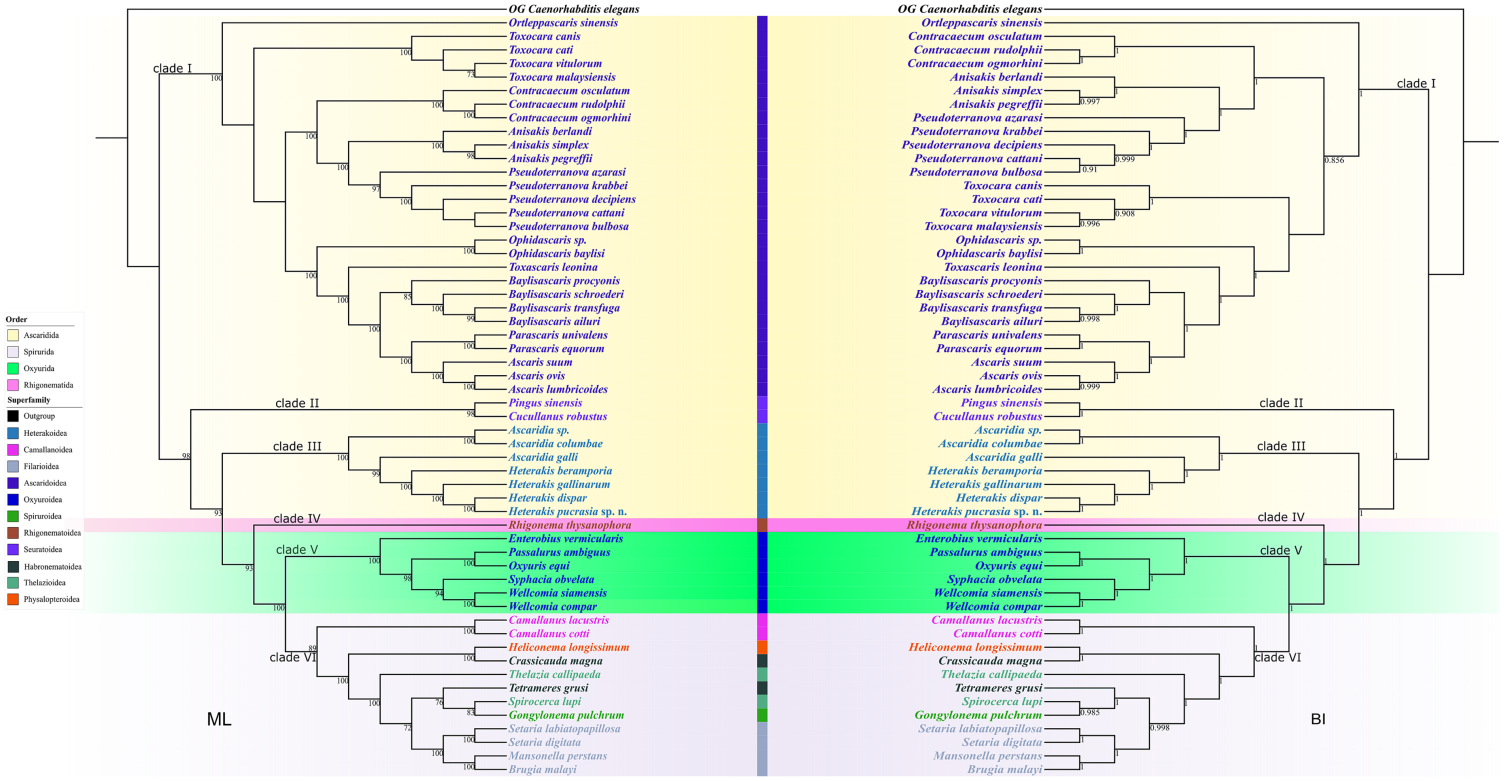
Phylogenetic results based on the concatenated amino acid sequences of 12 PCGs of mitogenomes of Ascaridida, Spirurida, Oxyurida and Rhigonematida. *Caenorhabditis elegans* (Rhabditida: Rhabditidae) was chosen as the out-group. Bootstrap values ≥70 and Bayesian posterior probability values ≥0.85 were shown in the phylogenetic trees.

Phylogenetic results based on the concatenated amino acid sequences of the 12 protein-coding genes of mitogenomes using BI and ML methods, are nearly identical, which showed the representatives of Ascaridida, Spirurida, Oxyurida and Rhigonematida divided into six large clades (Clade I-VI) (Figure 9). Clade I included species of the superfamily Ascaridoidea. Clade II included two representatives of the superfamily Seuratoidea (*Pingus sinensis* and *Cucullanus robustus*). Clade III contained species of *Ascaridia* and *Heterakis*, representing the superfamily Heterakoidea. The single representative *Rhigonema thysanophora* comprised the Clade IV, representing the order Rhigonematida. Clade V included six species of the order Oxyurida. Clade VI contained 12 species of the order Spirurida (Table 2).

**Table 2:** Base composition and skewness of mitogenome of *Heterakis pucrasia* sp. n. (PQ389430).

Location	A (%)	T (%)	C (%)	G (%)	AT (%)	AT-skew	GC-skew	Total (bp)
Whole mitochondrial genome	22.69	46.19	8.44	22.68	68.88	−0.34	0.46	13,978
Protein coding genes (PCGs)	20.49	47.78	8.50	23.23	68.27	−0.40	0.46	10,277
1st codon	27.22	39.86	8.78	24.13	67.08	−0.19	0.47	3,427
2nd codon	19.00	49.96	12.93	18.12	68.95	−0.45	0.17	3,427
3rd codon	15.26	53.52	3.79	27.43	68.78	−0.56	0.76	3,427
tRNAs	30.12	40.87	7.67	21.34	70.99	−0.15	0.47	1,265
rRNAs	27.62	43.73	7.15	21.50	71.35	−0.23	0.50	1,651
l-rRNA	25.89	47.17	5.97	20.96	73.06	−0.29	0.56	954
s-rRNA	29.99	39.02	8.75	22.24	69.01	−0.13	0.44	697
LNCR	29.24	39.10	13.41	18.26	68.34	−0.14	0.15	619
SNCR	37.40	34.96	8.13	19.51	72.36	0.03	0.41	123

## 4 Discussion

In the genus *Heterakis*, there have been about 38 nominal species reported from birds (26, 27). However, the validity of some species remains under debate. Among these species parasitic in birds, the new species is similar to the following species by having nearly equal spicule length with 0.50–1.00 mm, including *H. alata* Schneider, 1866, *H. skrjabini* Cram, 1927, *H. papillosa* (Bloch, 1782), *H. macroura* Linstow, 1883, *H. dispar* (Schrank, 1790) and *H. psophiae* Travassos, 1913. *Heterakis pucrasia* sp. n. differs from *H. psophiae* by having much larger precloacal sucker (0.19–0.20 mm in diameter in *H. pucrasia vs* only 0.09 mm in diameter in the latter) and longer male tail (0.65–0.66 mm in the new species *vs* 0.35 mm in *H. psophiae*) (28, 29). *Heterakis papillosa* is a poorly known heterakid nematode reported from *Otis tarda*, *O. tetrax* and *Tetrastes bonasia* in Europe and Asia (28, 30), which is very similar to the new species in the lengths of body and esophagus, the morphology and size of precloacal sucker, and the morphology and lengths of spicules and tail. However, the new species can be distinguished from *H. papillosa* by more caudal papillae (12 pairs in *H. pucrasia vs* 10 pairs in *H. papillosa*) and the different position of vulva (vulva 4.47–6.95 mm from cephalic extremity, representing 43.3–53.5% of body length in the new species *vs* vulva 6.29–8.12 mm from cephalic extremity, representing about 62.2% of body length in *H. papillosa*).

The new species differs from *H. dispar* by having distinctly longer male tail (0.65–0.66 mm in *H. pucrasia vs* about 0.32 mm in *H. dispar*), smaller female body (10.3–14.2 mm long in the new species *vs* 15.0–23.0 mm long in *H. dispar*) and the different position of vulva (the length of vulva from cephalic extremity representing 43.3–53.5% of body length in the new species *vs* representing about 56.3% of body length in *H. dispar*) (28, 29).

With the larger male body (17.0–27.8 mm), smaller posterior esophageal bulb (0.18–0.28 × 0.21–0.23 mm) and shorter male tail (0.28–0.37 mm), *H. alata* can be easily distinguished from the new species (male 9.10–9.39 mm long), posterior esophageal bulb 0.36–0.37 × 0.29–0.30 mm, male tail 0.65–0.66 mm (28, 31).

*Heterakis skrjabini* is also different from *H. pucrasia* sp. n. by having male tail lacking long filamentous tip (*vs* the presence of long filamentous tip in male in the new species), relatively shorter esophagus of female (esophageal length representing only 4.50% of body length in *H. skrjabini vs* representing 11.3–13.9% of body length in *H. pucrasia*) and the different position of the vulva (length of the vulva from cephalic extremity representing 17.7% of body length in *H. skrjabini vs* representing 43.3–53.5% of body length in the new species). *Heterakis macroura* is a common nematode parasite from birds of *Tetraogallus*.

Barus and Sonin (32) listed *H. altaica* Spaul, 1929 and *H. lahulensis* Soota & Chaturvedi, 1969 as synonyms of *H. macroura*. *Heterakis pucrasia* sp. n. differs from *H. macroura* by having more caudal papillae (14 pairs in the new species *vs* 12 pairs in *H. macroura*), more cuticular protrusions at region of vulva (presence of 4 or 5 cuticular protrusions in *H. pucrasia vs* usually presence of 2 cuticular protrusions in the latter) and smaller body of female (10.3–14.2 mm long in the new species *vs* 15.0–20.2 mm long in *H. macroura*) (28, 29).

Comparative mitogenome analysis revealed that the size and overall A+T content in the mitogenome of *H. pucrasia* sp. n. (13,978 bp in length, 68.9% overall A+T content) are similar to that of *H. gallinarum*, *H. beramporia* and *H. dispar* (13973–14,012 bp in length, and 69.8–71.3% overall A+T content in the latter three species) (13, 14). Moreover, the composition and gene arrangement of the mitogenome of *H. pucrasia* sp. n. are accordant with that of *H. gallinarum*, *H. beramporia* and *H. dispar*. To date, a total of 63 types of gene arrangement in the mitogenomes of Nematoda have been reported (33). The gene arrangement of the new species belongs to the GA1 type proposed by Yatawara et al. (34), which is identical to that of heterakoid species, including *H. gallinarum*, *H. beramporia*, *H. dispar*, *Ascaridia columbae*, *A. galli* and *Ascaridia* sp. (13, 14, 35).

Blaxter et al. (36) and Holterman et al. (37) provided phylogenies of the phylum Nematoda based on 18S sequence data, and preliminary revealed the evolutionary relationships of the four orders Ascaridida, Spirurida, Oxyurida and Rhigonematida. However, the phylogenetic relationships of these four orders, together with some their included superfamilies and families, remain uncertain (38). The present phylogenetic results showed that the current order Ascaridida is not monophyletic, which agreed well with some previous phylogenies (13, 35, 37–42). To date, only some molecular phylogenies tried to investigate the systematic position of the superfamily Heterakoidea, but most of phylogenetic results showed the Heterakoidea has a close relationship with the order Rhigonematida (37, 40–42); in contrast, some other results indicated the Heterakoidea has a close affinity with Ascaridoidea (38, 43, 44) or Oxyurida (39) or Rhabditomorpha + Ascaridomorpha (13, 35). Interestingly, the present phylogenetic results displayed the Heterakoidea clustered together with the representatives of Rhigonematida + Oxyurida + Spirurida, which are different from all of the previous studies (37, 38, 40–44). The present results suggested that the traditional superfamily Heterakoidea could be elevated as the infra-order Heterakomorpha. Although the present study provided novel insight into the systematic position of the Heterakoidea, it is yet an unsolved mystery of the phylogenetic position of the Heterakoidea. However, a more rigorous molecular phylogenetic study that includes broader representatives of the Heterakoidea, especially species of the families Aspidoderidae and Kiwinematidae, is needed to further solve the deep phylogenetic relationships between the Heterakoidea and its related groups.

## 5 Conclusion

A new species of *Heterakis*, *H. pucrasia* sp. n. was described using integrated methods based on specimens collected from the koklass pheasant *P. macrolopha* in Pakistan. The complete mitochondrial genome of *H. pucrasia* sp. n. was sequenced and annotated for the first time to enrich the mitogenomic data and reveal the pattern of mitogenomic evolution of Heterakidae. Mitogenomic phylogenetic results revealed that the order Ascaridida is not monophyletic, and suggested the erection of the infraorder Heterakomorpha for the traditional superfamily Heterakoidea.

## Data Availability

The nuclear and mitochondrial DNA sequences of *Heterakis pucrasia* sp. n. obtained in the present study were deposited in the National Center for Biotechnology Information (NCBI) database (http://www.ncbi.nlm.nih.gov) under the accession numbers: PQ373346, PQ373350, PQ358945 (18S); PQ358944, PQ358946, PQ358947 (28S); PQ373345 (ITS); PQ355460-PQ355462 (*cox1*); PQ374991, PQ373351, PQ374990 (12S); PQ365672-PQ365674 (*cox2*); PQ389430 (mitogenome).

## Footnotes

^1^ https://avibase.bsc-eoc.org/species.jsp?avibaseid=6383E214

^2^ http://www.ncbi.nlm.nih.gov

^3^ https://bioinfo.mnhn.fr/abi/public/asap/

^4^ http://mitos2.bioinf.uni-leipzig.de/index.py

^5^ https://www.ncbi.nlm.nih.gov/orffinder/
