# Supplementary material for: Morphology, complete mitochondrial genome and molecular phylogeny of Heterakis pucrasia sp. n. (Nematoda: Ascaridida) from the koklass pheasant Pucrasia macrolopha (Lesson) (Galliformes: Phasianidae) in Pakistan
Source: Front Vet Sci. 2025 Mar 3;12:1519092. doi: 10.3389/fvets.2025.1519092 (PMC11911484; doi:10.3389/fvets.2025.1519092)
Supplement: Supplementary file 1 [file Table_1.DOCX]

**Supplementary Table 1:** The primers and cycling conditions for amplifying different target regions using polymerase chain reaction (PCR) in the present study*.*

| Primer | Sequence 5'-3' | Cycling condition | References |
| --- | --- | --- | --- |
| 28S | 28S_HF1: 5′ - CTCGCTTCACATTACAGCCC - 3′  28S_HR1: 5′ - ACGTGCAAATCGATCGTCTG - 3′ | 94 °C for 5 min  94 °C for 30 s  59 °C for 30 s  72 °C for the 70 s (35 cycles)  72 °C for 7 min | present study |
| *cox*1 | NemF1 t1: 5′ - CRACWGTWAATCAYAARAATATTGG - 3′  NemF2 t1: 5′ - ARAGATCTAATCATAAAGATATYGG - 3′  NemF3 t1: 5′ - ARAGTTCTAATCATAARGATATTGG - 3′  NemR1 t1: 5′ - AAACTTCWGGRTGACCAAAAAATCA - 3′  NemR2 t1: 5′ - AWACYTCWGGRTGMCCAAAAAAYCA - 3′  NemR3 t1: 5′ - AAACCTCWGGATGACCAAAAAATCA - 3′ | 95 °C for 1 min  95 °C for 40 s  45 °C for 40 s  72 °C for 1 min (5 cycles)  94 °C for 40 s  51 °C for 40 s  72°C for 1min (35 cycles)  72°C for 5 min | (1) |
| 12S | 12S-F: 5′ - GTTCCAGAATAATCGGCTA - 3′  12S-R: 5′ - ATTGACGGATGGTTTGTACC - 3′ | 94°C for 5 min  94 °C for 30 s  55 °C for 30 s  72 °C for 45 s (35 cycles)  72 °C for 7 min | (2, 3) |
| *cox*2 | cox2_HF1: 5′ - TGGGGTTTTGCTGTTTGTAAGT - 3′  cox2_HR1: 5′ - CCCCGCAAATCTCAGAACAC - 3′ | 95 °C for 15 min  95 °C for 60 s  59 °C for 90 s  72°C for 75 s (35 cycles)  72°C for 7 min | present study |
| 18S | 18S_F: 5′ - CGCGAATRGCTCATTACAACAGC - 3′  18S_R: 5′ - GGGCGGTATCTGATCGC - 3′ | 94 °C for 5 min  94 °C for 30 s  55 °C for 30 s  72 °C for 70 s (35 cycles)  72°C for 7 min | (3, 4) |

**Supplementary Table 2.** Detailed information on the representatives of the orders Ascaridida, Spirurida, Oxyurida and Rhigonematida included in the present phylogenetic analyses.

| Species | Accession nos. | Length (bp) | AT (%) | References |
| --- | --- | --- | --- | --- |
| **Ingroup**  **Ascaridida**  **Heterakoidea** |  |  |  |  |
| **Ascaridiidae** |  |  |  |  |
| *Ascaridia columbae* | NC_021643.1 | 13931 | 71.1 | (5) |
| *Ascaridia* sp. | JX624730.1 | 13862 | 67.1 | (5) |
| *Ascaridia galli* | OQ286042.1 | 13981 | 71.6 | Unpublished |
| **Heterakidae** |  |  |  |  |
| *Heterakis beramporia* | KU529972.1 | 14012 | 71.3 | (6) |
| *Heterakis dispar* | NC_042411.1 | 13995 | 69.8 | (7) |
| *Heterakis gallinarum* | NC_029839.1 | 13973 | 69.8 | (6) |
| *Heterakis pucrasia* sp. n. | PQ389430 | 13978 | 68.9 | This study |
| **Ascaridoidea** |  |  |  |  |
| **Anisakidae** |  |  |  |  |
| *Anisakis pegreffii* | NC_034329.1 | 14002 | 71.3 | (8) |
| *Anisakis berlandi* | NC_026023.1 | 13915 | 71.3 | Unpublished |
| *Anisakis simplex* | KU899549.1 | 13938 | 71.4 | Unpublished |
| *Pseudoterranova decipiens* | NC_031645.1 | 13962 | 71.1 | Unpublished |
| *Pseudoterranova cattani* | NC_031644.1 | 13950 | 71.1 | Unpublished |
| *Pseudoterranova bulbosa* | NC_031643.1 | 13957 | 71.2 | Unpublished |
| *Pseudoterranova azarasi* | NC_027163.1 | 13954 | 70.7 | Unpublished |
| *Pseudoterranova krabbei* | KU558724.1 | 13948 | 70.4 | Unpublished |
| *Contracaecum ogmorhini* | NC_031649.1 | 14010 | 71.2 | Unpublished |
| *Contracaecum osculatum* | NC_024037.1 | 13823 | 70.2 | (9) |
| *Contracaecum rudolphii* | NC_014870.1 | 14022 | 70.4 | Unpublished |
| *Ophidascaris* sp. | MK106624.1 | 14660 | 69.3 | (10) |
| *Ophidascaris baylisi* | MW880927.1 | 14784 | 70 | (11) |
| **Ascarididae** |  |  |  |  |
| *Baylisascaris procyonis* | JF951366.1 | 14781 | 70.5 | (12) |
| *Baylisascaris schroederi* | HQ671081.1 | 14778 | 68.6 | (13) |
| *Baylisascaris ailuri* | HQ671080.1 | 14657 | 69.5 | (13) |
| *Baylisascaris transfuga* | HQ671079.1 | 14898 | 69.4 | (13) |
| *Ortleppascaris sinensis* | KU950438.1 | 13828 | 74.1 | (14) |
| *Toxascaris leonina* | MK516267.1 | 14685 | 71.1 | Unpublished |
| *Parascaris equorum* | MF678786.1 | 13899 | 70.2 | Unpublished |
| *Parascaris univalens* | NC_024884.1 | 13920 | 70.6 | (15) |
| *Ascaris lumbricoides* | NC_016198.1 | 14281 | 71.9 | (16) |
| *Ascaris suum* | NC_001327.1 | 14284 | 72 | (17) |
| *Ascaris ovis* | MT993838.1 | 14205 | 71.9 | (18) |
| **Toxocaridae** |  |  |  |  |
| *Toxocara canis* | NC_010690.1 | 14322 | 68.6 | (19) |
| *Toxocara vitulorum* | NC_070176.1 | 15045 | 70 | (20) |
| *Toxocara cati* | NC_010773.1 | 14029 | 70 | (19) |
| *Toxocara malaysiensis* | NC_010527.1 | 14266 | 68.9 | (19) |
| **Seuratoidea**  **Quimperiidae** |  |  |  |  |
| *Pingus sinensis* | MW971502.1 | 13874 | 68.7 | (21) |
| **Cucullanidae** |  |  |  |  |
| *Cucullanus robustus* | NC_016128.1 | 13972 | 71.6 | (22) |
| **Oxyurida**  **Oxyuroidea**  **Oxyuridae** |  |  |  |  |
| *Wellcomia compar* | MW059037.1 | 14373 | 78.7 | (23) |
| *Wellcomia siamensis* | NC_016129.1 | 14128 | 77.9 | (22) |
| *Passalurus ambiguus* | NC_028345.1 | 14023 | 71.6 | (24) |
| *Oxyuris equi* | NC_027190.1 | 13641 | 67.8 | (25) |
| *Enterobius vermicularis* | EU281143.1 | 14010 | 71.2 | (26) |
| *Syphacia obvelata* | KT900946.1 | 14231 | 74.1 | (27) |
| **Rhigonematida**  **Rhigonematoidea**  **Rhigonematidae** |  |  |  |  |
| *Rhigonema thysanophora* | NC_024020.1 | 15015 | 67.7 | (28) |
| **Spirurida**  **Camallanoidea**  **Camallanidae** |  |  |  |  |
| *Camallanus cotti* | NC_036308.1 | 17901 | 70.8 | (29) |
| *Camallanus lacustris* | OM469016.1 | 18935 | 75.9 | (30) |
| **Habronematoidea**  **Tetrameridae** |  |  |  |  |
| *Crassicauda magna* | OQ834322.1 | 13604 | 74.9 | (31) |
| *Tetrameres grusi* | NC_062325.1 | 13709 | 71.6 | (32) |
| **Thelazioidea**  **Thelaziidae** |  |  |  |  |
| *Thelazia callipaeda* | KY908320.1 | 13666 | 74.8 | (33) |
| *Spirocerca lupi* | MK922357.1 | 13804 | 74.4 | (34) |
| **Filarioidea**  **Onchocercidae** |  |  |  |  |
| *Brugia malayi* | MT149211.1 | 13658 | 75.6 | (35) |
| *Mansonella perstans* | NC_077638.1 | 13616 | 74.1 | (36) |
| **Setariidae** |  |  |  |  |
| *Setaria labiatopapillosa* | MH937750.1 | 13950 | 78.9 | (37) |
| *Setaria digitata* | KY284626.1 | 13814 | 74.9 | (38) |
| **Spiruroidea**  **Gongylonematidae** |  |  |  |  |
| *Gongylonema pulchrum* | NC_026687.1 | 13798 | 76 | (39) |
| **Physalopteroidea**  **Physalopteridae** |  |  |  |  |
| *Heliconema longissimum* | GQ332423.1 | 13610 | 79.1 | (22) |
| **Outgroup** |  |  |  |  |
| **Rhabditida**  **Rhabditoidea**  **Rhabditidae** |  |  |  |  |
| *Caenorhabditis elegans* | NC_001328.1 | 13794 | 76.2 | (17) |

**References**

1. Prosser SW, Velarde‐Aguilar MG, León‐Règagnon V, Hebert PD. Advancing nematode barcoding: a primer cocktail for the cytochrome c oxidase subunit I gene from vertebrate parasitic nematodes. *Mol Ecol Resour.* (2013) 13:1108–15.

2. Li Y, Niu L, Wang Q, Zhang Z, Chen Z, Gu X, et al. Molecular characterization and phylogenetic analysis of ascarid nematodes from twenty-one species of captive wild mammals based on mitochondrial and nuclear sequences. *Parasitology.* (2012) 139:1329–38.

3. Li L, Lü L, Nadler SA, Gibson DI, Zhang L-P, Chen H-X, et al. Molecular phylogeny and dating reveal a terrestrial origin in the early carboniferous for ascaridoid nematodes. *Syst Biol.* (2018) 67:888–900.

4. Floyd RM, Rogers AD, Lambshead PJD, Smith CR. Nematode‐specific PCR primers for the 18S small subunit rRNA gene. *Mol Eco Notes.* (2005) 5:611–2.

5. Liu G-H, Shao R, Li J-Y, Zhou D-H, Li H, Zhu X-Q. The complete mitochondrial genomes of three parasitic nematodes of birds: a unique gene order and insights into nematode phylogeny. *BMC Genom.* (2013) 14:1-13.

6. Wang B-J, Gu X-B, Yang G-Y, Wang T, Lai W-M, Zhong Z-J, et al. Mitochondrial genomes of *Heterakis gallinae* and *Heterakis beramporia* support that they belong to the infraorder Ascaridomorpha. *Infect Genet Evol.* (2016) 40:228–35.

7. Gao J-F, Hou M-R, Wang W-F, Gao Z-Y, Zhang X-G, Lu Y-X, et al. The complete mitochondrial genome of *Heterakis dispar* (Ascaridida: Heterakidae). *Mitochondrial DNA B*. (2019) 4:1630-1.

8. Yamada A, Ikeda N, Ono H. The complete mitochondrial genome of *Anisakis pegreffii* Campana-Rouget & Biocca, 1955,(Nematoda, Chromadorea, Rhabditida, Anisakidae)–clarification of mitogenome sequences of the *Anisakis simplex* species complex. *Mitochondrial DNA Part.* (2017) 2:240–1.

9. Mohandas N, Jabbar A, Podolska M, Zhu X-Q, Littlewood DTJ, Jex AR, et al. Mitochondrial genomes of *Anisakis simplex* and *Contracaecum osculatum* (sensu stricto)–comparisons with selected nematodes. *Infect Genet Evol.* (2014) 21:452–62.

10. Han L, Yang Y, Li H, Zhou X, Zhou M, Liu T, et al. Gene rearrangements in the mitochondrial genome of ten ascaris species and phylogenetic implications for Ascaridoidea and Heterakoidea families. *Int J Biol Macromol.* (2022) 221:1394–403.

11. Zhao Q, Abuzeid AM, He L, Zhuang T, Li X, Liu J, et al. The mitochondrial genome sequence analysis of *Ophidascaris baylisi* from the Burmese python (*Python molurus bivittatus*). *Parasitol Int.* (2021) 85:e102434. doi：10.1016/j.parint.2021.102434

12. Xie Y, Zhang Z, Niu L, Wang Q, Wang C, Lan J, et al. The mitochondrial genome of *Baylisascaris procyonis.* *PloS One.* (2011) 6:e27066. doi. 10.1371/journal.pone.0027066

13. Xie Y, Zhang Z, Wang C, Lan J, Li Y, Chen Z, et al. Complete mitochondrial genomes of *Baylisascaris schroederi*, *Baylisascaris ailuri* and *Baylisascaris transfuga* from giant panda, red panda and polar bear. *Genes.* (2011) 482:59–67.

14. Zhao J, Tu G, Wu X, Li C. Characterization of the complete mitochondrial genome of *Ortleppascaris sinensis* (Nematoda: Heterocheilidae) and comparative mitogenomic analysis of eighteen Ascaridida nematodes. *J Helminthol.* (2018) 92:369–78.

15. Jabbar A, Littlewood DTJ, Mohandas N, Briscoe AG, Foster PG, Müller F, et al. The mitochondrial genome of *Parascaris univalens*-implications for a “forgotten” parasite. *Parasite Vector.* (2014) 7:1–8.

16. Park YC, Kim W, Park J-K. The complete mitochondrial genome of human parasitic roundworm, *Ascaris lumbricoides*. *Mitochondrial DNA.* (2011) 22:91–3.

17. Okimoto R, Macfarlane J, Clary D, Wolstenholme D. The mitochondrial genomes of two nematodes, *Caenorhabditis elegans* and *Ascaris suum*. *Genetics*. (1992) 130:471-98.

18. Chen Y, Wang L, Zhou X, Tang R, Li Y, Liu Y, et al. The mitochondrial genome of the sheep roundworm *Ascaris ovis* (Ascaridida: Nematoda) from Southwest China. *Mitochondrial DNA B.* (2021) 6:410–2.

19. Li M-W, Lin R-Q, Song H-Q, Wu X-Y, Zhu X-Q. The complete mitochondrial genomes for three *Toxocara* species of human and animal health significance. *BMC Genomics.* (2008) 9:1–10.

20. Xie Y, Wang L, Chen Y, Wang Z, Zhu P, Hu Z, et al. The complete mitogenome of *Toxocara vitulorum*: novel in-sights into the phylogenetics in Toxocaridae. *Animals*. (2022) 12:e3546. doi: 10.3390/ani12243546

21. Chen F, Zou H, Jin X, Zhang D, Li W, Li M, et al. Sequencing of the complete mitochondrial genome of *Pingus sinensis* (Spirurina: Quimperiidae): Gene arrangements and phylogenetic implications. *Genes.* (2021) 12:e1772. doi: 10.3390/genes12111772

22. Park J-K, Sultana T, Lee S-H, Kang S, Kim HK, Min G-S, et al. Monophyly of clade III nematodes is not supported by phylogenetic analysis of complete mitochondrial genome sequences. *BMC Genomics.* (2011) 12:1–16.

23. Huo C, Bao F, Long H, Qin T, Zhang S. The complete mitochondrial genome of *Wellcomia compar* (Spirurina: Oxyuridae) and its genome characterization and phylogenetic analysis. *Sci Rep.* (2023) 13:e14426. doi: 10.1038/s41598-023-41638-9

24. Liu G-H, Li S, Zou F-C, Wang C-R, Zhu X-Q. The complete mitochondrial genome of rabbit pinworm *Passalurus ambiguus*: genome characterization and phylogenetic analysis. *Parasitol Res.* (2016) 115:423–9.

25. Zhang Y, Xu W-W, Guo D-H, Liu Z-X, Duan H, Su X, et al. The complete mitochondrial genome of *Oxyuris equi:* comparison with other closely related species and phylogenetic implications. *Exp Parasitol.* (2015) 159:215–21.

26. Kang S, Sultana T, Eom KS, Park YC, Soonthornpong N, Nadler SA, et al. The mitochondrial genome sequence of *Enterobius vermicularis* (Nematoda: Oxyurida)—an idiosyncratic gene order and phylogenetic information for chromadorean nematodes. *Genes.* (2009) 429:87–97.

27. Wang C-R, Lou Y, Gao J-F, Qiu J-H, Zhang Y, Gao Y, et al. Comparative analyses of the complete mitochondrial genomes of the two murine pinworms *Aspiculuris tetraptera* and *Syphacia obvelata.* *Genes.* (2016) 585:71–5.

28. Kim T, Kim J, Cho S, Min GS, Park C, Carreno RA, et al. Phylogeny of Rhigonematomorpha based on the complete mitochondrial genome of *Rhigonema thysanophora* (Nematoda: Chromadorea). *Zool Scr.* (2014) 43:289–303.

29. Zou H, Jakovlić I, Chen R, Zhang D, Zhang J, Li W-X, et al. The complete mitochondrial genome of parasitic nematode *Camallanus cotti*: extreme discontinuity in the rate of mitogenomic architecture evolution within the Chromadorea class. *BMC Genomics.* (2017) 18:1–17.

30. Zou H, Lei H-P, Chen R, Chen F-L, Li W-X, Li M, et al. Evolutionary rates of mitochondrial sequences and gene orders in Spirurina (Nematoda) are episodic but synchronised. *Water Biol Secur.* (2022) 1:100033.

31. Qiao Y, Ma X, Zhong S, Xing Y, Chen X, Chen B. The first complete mitochondrial genome of macroparasite *Crassicauda magna* (Nematoda: Spirurida) from *Neophocoena sunameri* in ningbo, China. *Mitochondrial DNA B.* (2021) 6:3011–2.

32. Gao J-F, Mao R-F, Li Y, Sun Y-Y, Gao Z-Y, Zhang X-G, et al. Characterization of the mitochondrial genome of *Tetrameres grusi* and insights into the phylogeny of Spirurina. *Int J Parasitol-PAW.* (2022) 17:35–42.

33. Zhang X, Shi YL, Wang ZQ, Duan JY, Jiang P, Liu RD, et al. Morphological and mitochondrial genomic characterization of eyeworms (*Thelazia callipaeda*) from clinical cases in central China. *Front Microbiol.* (2017) 8:e1335.

34. Rothmann-Meyer W, Naidoo K, de Waal PJ. Comparative mitogenomics of *Spirocerca lupi* from South Africa and China: variation and possible heteroplasmy. *Vet Parasitol.* (2021) 300:e109595.

35. Qing X, Kulkeaw K, Wongkamchai S, Tsui SK-W. Mitochondrial genome of *Brugia malayi* microfilariae isolated from a clinical sample. *Front Ecol Evol.* (2021)9:e637805.

36. Rodi M, Gross C, Sandri TL, Berner L, Marcet-Houben M, Kocak E, et al. Whole genome analysis of two sympatric human Mansonella: *Mansonella perstans* and *Mansonella* sp. “DEUX”. *Front Cell Infect Microbiol.* (2023) 13:e1159814.

37. Gao J-F, Hou M-R, Cui Y-C, Shi T-R. The complete mitochondrial genome of *Setaria labiatepapillosa* (Spirurida: Setariidae). *Mitochondrial DNA B.* (2019) 4:1632–3.

38. Liu G-H, Li J-Y, Zhu X-Q. Characterization of the complete mitochondrial genome of *Setaria digitata* (Nematoda: Setariidae) from China. *J Helminthol.* (2017) 91:772–6.

39. Liu G-H, Jia Y-Q, Wang Y-N, Zhao G-H, Zhu X-Q. The complete mitochondrial genome of the gullet worm *Gongylonema pulchrum*: gene content, arrangement, composition and phylogenetic implications. *Parasite Vector*. (2015) 8:1–8.
